# Paediatric Acute Respiratory Distress Syndrome as the introductory manifestation in a patient with Childhood Onset Systemic Lupus Erythematosus

**DOI:** 10.31138/mjr.30.2.135

**Published:** 2019-06-29

**Authors:** Efstratia Kontaxi, Maria Trachana, Polyxeni Pratsidou-Gertsi, Eleni Volakli, Asimenia Violaki, Maria Sdougka

**Affiliations:** 1 Pediatric Immunology and Rheumatology Referral Center, First Department of Pediatrics, Aristotle University, Ippokration Hospital, Thessaloniki, Greece,; 2 Pediatric Intensive Care Unit, Ippokration Hospital, Thessaloniki, Greece

Dear Sir,

Acute Respiratory Distress Syndrome (ARDS) is a potentially life-threatening emergency within a spectrum of pulmonary diseases characterized by hypoxemia, non-cardiogenic pulmonary edema and a deregulated alveolar hyper-inflammation. The diagnosis-regardless of age-relies on the Berlin Definition criteria (BDC, *Table 1*). It usually complicates the disease course in patients with infections, malignancies and congenital or acquired immunodeficiencies, the latter including patients with SLE.^1^ Conversely, Childhood Onset SLE (cSLE) is associated with severe pulmonary manifestations, as pleuritis, pulmonary hypertension, acute lupus pneumonitis, chronic interstitial pneumonitis, alveolar hemorrhage, pulmonary embolism and shrinking lung syndrome.^2,3^ Due to the rareness of relevant publications in preadolescent patients, we report a diagnostic challenge, a surviving case of a severe pediatric ARDS (pARDS) which was the introductory manifestation of cSLE. The father of the patient described here provided his written consent for publication according to the Declaration of Helsinki. The patient’s management was according to standards of the SLE care.

**Table 1.** Berlin definition for acute respiratory distress syndrome.

Timing	Within 1 week of a known clinical insult or new or worsening respiratory symptoms
Chest imaging (radiograph or CT)	Bilateral opacities — not fully explained by effusions, lobar/lung collapse, or nodules
Origin of oedema	Respiratory failure not fully explained by cardiac failure or fluid overload. Need objective assessment (e.g., echocardiography) to exclude hydrostatic oedema if no risk factor present
Oxygenation^*^	Mild 200 mmHg < PaO2/FIO2 ≤300 mmHg with PEEP or CPAP ≥5 cmH2O^**^ Moderate 100 mmHg < PaO2/FIO2 ≤200 mmHg with PEEP ≥5 cmH2O Severe PaO2/FIO2 ≤100 mmHg with PEEP ≥5 cmH2O

CPAP, continuous positive airway pressure; FIO2, fraction of inspired oxygen; PaO2, partial pressure of arterial oxygen; PEEP, positive end-expiratory pressure.

* If altitude is higher than 1,000 m, the correction factor should be calculated as follows: [PaO2/FIO2 (barometric pressure/760)];

** This may be delivered noninvasively in the mild acute respiratory distress syndrome group.

**Figure 1.**
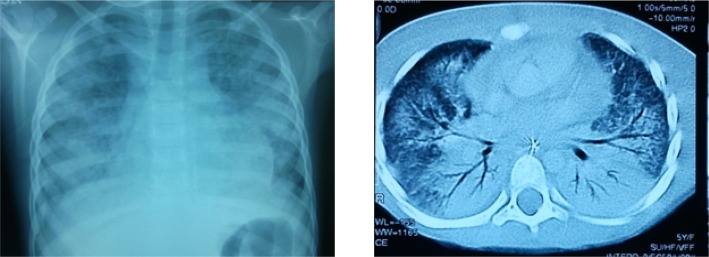
Reticulonodular infiltrates of middle and lower lung lobes, ground glass opacities (A, B) and interlobular septal thickening (B).

A 5-year intubated girl was transferred to the pediatric intensive care unit (PICU) due to a progressing febrile respiratory failure accompanied by micromacular malar rash, bilateral limb oedema, hepatosplenomegaly and generalized lymphadenopathy. The disease onset was 10-day ago with fever (Τ>38.5^°^ C), malaise and cough which progressed to a recalcitrant to several anti-infectious regimens pneumonia. She had normocytic anaemia (Hb 9g/dL), proteinuria (urine protein to creatinine ratio [p/cr] 0.67), ESR 67mm/h, CRP 40mg/L (normal<5 mg/L) without any pathology of the heart or bone marrow, and sterile biologic samples. Fundoscopy revealed papilledema with retinal haemorrhages and her lumbar puncture, intracranial hypertension. Radiology showed bilateral reticulonodular infiltrates of middle and lower lung lobes (*Figure 1a*), abdominal MRI displayed hepatosplenomegaly with retroperitoneal lymphadenopathy, and her brain MRI was normal.

She was treated with antibiotics, mechanical ventilation, inotropes and antihypertensives. According to the BDC (*Table 1*), she met all 4 criteria of ARDS: gradual worsening of respiratory symptoms, abnormal chest X-ray with bilateral opacities and respiratory failure with severely impaired oxygenation, with a previous normal echocardiography.^1^ The bronchoalveolar lavage (BAL) serial work-up did not have any RBCs or findings of diffuse alveolitis in any specimen and cultures were always sterile.

Consecutive blood, urine and gastric fluid cultures remained also sterile. However, her anaemia deteriorated (Hb 7.93g/dL) and coagulopathy emerged (PLT 66000/mm^3^ and prolonged aPTT). Immunology screening revealed IgG 34 g/L (normal 7–15 g/L), IgA 2.86 g/L (normal 0.5–1.8 g/L), IgM 2.39 g/L (normal 0.45–2.3 g/L, IgE 3.86) IU/mL (normal 8.3–121 IU/mL), C3 0.14 g/L (normal 0.9–1.8 g/L) and C4 0.02 g/L (normal 0.1–0.4 g/L), high titers of ANA (1/640, normal <1:160), anti-dsDNA (1/640, normal <1:20) and positive antiphospholipid antibodies: β2 GPI IgM and IgG (36 U/mL, and 25U/mL, normal<7U/mL).

According to the paediatric rheumatologist’s assessment, this was a cSLE case which fulfilled both ACR (6/11) and SLICC classification criteria (5/11 of clinical and 4/6 of immunologic criteria).^2^ The regimen was switched to intravenous methylprednisolone pulses (3 pulses, of 30mg/kg each) and cyclophosphamide (500mg/m^2^). Consecutive CTs showed lower lung lobes consolidations and “ground glass” opacities (*Figure 1b*), persistent generalized chest and abdominal lymphadenopathy and ascites. Echocardiography revealed left ventricle hypertrophy, pericarditis and mild pulmonary hypertension. Lymph node and bone marrow biopsies were negative for malignancy. After a 4-week care in PICU, the patient gradually stabilized, weaned off the mechanical ventilation and was transferred to the Paediatric Department.

Her respiratory distress subsided, and she became normotensive. However, renal histology revealed a Class II mesangial proliferative nephritis with IgG, IgA and IgM, C3d, C1q diffuse and moderate granular mesangial depositions, and her renal function was decreased. Eye examination showed persistent vascular twisting indicative of SLE vasculitis. Repeated CTs depicted resolution of pulmonary consolidations, pleural and abdominal effusions, but persistence of the enlarged pulmonary lymphadenopathy and persistent “ground glass” appearance. Complement levels remained low (C3 0.55 and C4 0.06 g/L), whereas ANA and anti-DNA titers (1/160 and 1/40), were decreased.

Prednisolone, cyclophosphamide, hydroxychloroquine, bronchodilators, antihypertensives and enoxaparin contributed further to her improvement. Anticoagulants were administered due to the presence of anticardiolipins in addition to the long - over a month - intubated stay in PICU. However, she had developed a mild obstructive pulmonary dysfunction and was treated with systemic inhalers, salmeterol plus fluticasone.

Six years later, under prednisolone 0.3mg/d, hydroxychloroquine, MMF and Belimumab, she contracted measles from her sibling, as she was unimmunized with MMR. She experienced another, although milder, episode of ARDS, that was successfully treated in PICU with mechanical ventilation, IV antibiotics, inotropes and bronchodilators. At her last evaluation, 6-month later, she had no further respiratory dysfunction and her eyes were free of any sequelae. Due the early disease onset, the possibility of monogenic lupus could not be ruled out, but genetic analysis could not be performed.

To our knowledge, this is among the first cases of severe pARDS, preceding (not following, as previously published) cSLE diagnosis.^2–5^ In contrast to previous publications, our case described a very young patient (5-year-old) who rapidly developed severe lupus pneumonitis within a short time of 10 days.^3,6,7^

It projected as a recalcitrant non-cardiogenic, progressively deteriorating pulmonary inflammation. Diagnosis of pARDS was established by revised BDC and of cSLE supported by ACR and SLICC criteria. Several deregulated inflammatory mechanisms triggered by infections, lung trauma or deranged immunity disrupt the alveolar capillary permeability and prevent extravascular oedema resorption. Resolution of ARDS requires the in-situ recruitment of immune “anti-inflammatory” cells and restoration of epithelial and endothelial integrity. Future delineation of individual host differences of inflammatory and cell signalling systems will lead to a targeted management of ARDS.^1,6–10^

Despite treatment, pARDS (estimated incidence 3.9/100.000 children) has a significant morbidity and mortality. Its severity, management and uneventful outcome are further impaired by immunodeficiencies, as was this cSLE case.^8–10^ Other affected sites, as this patient’s retinal vasculitis may rarely accompany the dramatic onset.^11^ The single most important independent clinical risk factor for mortality in pARDS’ onset is the multiple organ/system dysfunction.^8–10^

This particular case highlights that pARDS may be the invading and life-threatening manifestation “covering” an underlying rheumatic disease and arise with an unexplained and deteriorating alveolar failure. Additionally, the potential risk of recurrent pARDS in case of infections in these partially immunized patients, requires the physician’s vigilance for early and successful diagnosis and management. Noteworthy, the unusual incidence of cSLE onset prior to the age of 6 years, as in the presented patient, may have a genetic background, and research of mutations involved in monogenic lupus will be planned. ^12^ The rarity of lupus manifestations in young patients aligns with a recent Brazilian publication that included 847 SLE patients. The estimated incidence in patients less than 6 years was 4.6% and underlined the required physician’s vigilance for the existence of SLE in preschoolers.^13^

In conclusion and to our knowledge, this case described the youngest survivor of severe ARDS, with a very short disease onset and a rapidly progressing dramatic pneumonitis that led to the diagnosis of cSLE.
